# Longitudinal characterization of nasopharyngeal colonization with *Streptococcus pneumoniae* in a South African birth cohort post 13-valent pneumococcal conjugate vaccine implementation

**DOI:** 10.1038/s41598-018-30345-5

**Published:** 2018-08-21

**Authors:** Felix S. Dube, Jordache Ramjith, Sugnet Gardner-Lubbe, Polite Nduru, F. J. Lourens Robberts, Nicole Wolter, Heather J. Zar, Mark P. Nicol

**Affiliations:** 1 Department of Molecular and Cell Biology, Faculty of Science, University of Cape Town, Cape Town, South Africa; 2 Division of Epidemiology & Biostatistics, School of Public Health & Family Medicine, University of Cape Town, Cape Town, South Africa; 3 Department of Statistical Sciences, Faculty of Science, University of Cape Town, Cape Town, South Africa; 4 Centre for Respiratory Diseases and Meningitis (CRDM), National Institute for Communicable Diseases of the National Health Laboratory Service, Johannesburg, South Africa; 5 School of Pathology, Faculty of Health Sciences, University of the Witwatersrand, Johannesburg, South Africa; 6 Department of Paediatrics and Child Health, Red Cross War Memorial Children’s Hospital, University of Cape Town, Cape Town, South Africa; 7 SAMRC Unit on Child and Adolescent Health, University of Cape Town, Cape Town, South Africa; 8 Division of Medical Microbiology, Department of Pathology, Faculty of Health Sciences, University of Cape Town, Cape Town, South Africa; 9 National Health Laboratory Service, Groote Schuur Hospital, Cape Town, South Africa; 10 Institute for Infectious Diseases and Molecular Medicine, Faculty of Health Sciences, University of Cape Town, Cape Town, South Africa

## Abstract

Monitoring changes in pneumococcal carriage is key to understanding vaccination-induced shifts in the ecology of carriage and impact on health. We longitudinally investigated pneumococcal carriage dynamics in infants. Pneumococcal isolates were obtained from nasopharyngeal (NP) swabs collected 2-weekly from 137 infants enrolled from birth through their first year of life. Pneumococci were serotyped by sequetyping, confirmed by Quellung. Pneumococci were isolated from 54% (1809/3331) of infants. Median time to first acquisition was 63 days. Serotype-specific acquisition rates ranged from 0.01 to 0.88 events/child-year and did not differ between PCV13 and non-PCV13 serotypes (0.11 events/child-year [95% CI 0.07–0.18] *vs*. 0.11 events/child-year [95% CI 0.06–0.18]). There was no difference in carriage duration between individual PCV13 and non-PCV13 serotypes (40.6 days [95% CI 31.9–49.4] *vs*. 38.6 days [95% CI 35.1–42.1]), however cumulatively the duration of carriage of non-PCV13 serotypes was greater than PCV13 serotypes (141.2 days (95% CI 126.6–155.8) vs. 30.7 days (95% CI 22.3–39.0). Frequently carried PCV13 serotypes included 19F, 9V, 19A and 6A, while non-PCV13 serotypes included 15B/15C, 21, 10A, 16F, 35B, 9N and 15A. Despite high immunization coverage in our setting, PCV13 serotypes remain in circulation in this cohort, comprising 22% of isolates. Individual PCV13 serotypes were acquired, on average, at equivalent rate to non-PCV13 serotypes, and carried for a similar duration, although the most common non-PCV13 serotypes were more frequently acquired than PCV13 serotypes.

## Introduction

*Streptococcus pneumoniae* (pneumococcus) is a leading bacterial cause of upper respiratory infections such as otitis media and sinusitis, and severe disease such as pneumonia, sepsis and meningitis^1,2^. Although there have been substantial reductions in overall child mortality and in pneumonia-specific mortality, childhood pneumonia remains the major single cause of death in children outside the neonatal period, causing approximately 900,000 of the estimated 6.3 million global deaths in children under 5 years of age in 2013^3^. The incidence of pneumonia-related mortality is particularly high in Africa with more than 600,000 pneumonia-related deaths reported in children each year^4^.

Nasopharyngeal (NP) colonization by pneumococci is a necessary first step in progression to pneumococcal pneumonia and yet the dynamic nature of colonization remains incompletely understood. Children under the age of two years are more frequently colonized by pneumococci than older children and adults^5^. The nasopharynx also serves as a reservoir and source for transmission of pneumococci^6^.

The pneumococcal capsular polysaccharide is an important virulence determinant. There are over 98 different pneumococcal serotypes, characterized by an antigenic polysaccharide capsule with differing invasive disease potential, and elicits type-specific immunity^7^. Pneumococcal serotypes also differ in prevalence and extent of antibiotic resistance^7,8^. Prevention of NP colonization in children is a strategy to prevent pneumococcal disease in the vaccinated child as well as in others (herd protection due to reduced transmission). Vaccination with the pneumococcal conjugate vaccine (PCV; the current vaccine used in South Africa [PCV13], targets 13 serotypes) is effective in preventing serotype-specific invasive disease (IPD). More specifically, pre-PCV (2005–2008) and post-PCV (2012–2013) data from South Africa has shown a 62% reduction in all serotype IPD from 107, 600 (95% CI 83,000–140,000) to 41, 800 (95% CI 28,000–50,000) cases of severe hospitalised pneumococcal disease in children aged 0–59 months^9^. This was mirrored by a decline in colonization prevalence from 66.4% to 56.6% (odds ratio [OR] = 0.66, 95% CI: 0.44–0.98) for all serotypes, 30.4% to 9.6% (OR = 0.24, 95% CI: 0.16–0.38) for PCV7 serotypes, and 10.4% to 4.2% (OR = 0.38, 95% CI: 0.19, 0.73) for PCV13-additional serotypes. In contrast, the non–PCV colonization prevalence increased from 25.6% to 42.9% by 2012 (OR = 2.18, 95% CI: 1.42, 3.34)^10^. The overall prevalence of the pneumococcus have remained largely unchanged due to the replacement of PCV13 serotypes with emerging non-PCV13 serotypes seen in both colonized and infected vaccinated patients^11^.

The duration of pneumococcal carriage varies across epidemiological settings, with the median duration ranging from 60 days (serotype 11A) to 212 days (serotype 19F) when sampled at monthly intervals^12^. Recent acquisition of pneumococci in the NP has been associated with progression to disease^13^.

Pneumococcal carriage dynamics are best understood from longitudinal rather than cross-sectional studies^12,14,15^. Longitudinal study designs allow for the estimation of acquisition rates, carriage duration and risk factors with optimized sampling frequency, length of follow-up and clinical data collection^12^. We aim to describe the dynamics of pneumococcal NP carriage during the first year of life in an intensively sampled PCV13-vaccinated paediatric birth cohort in South Africa.

## Materials and Methods

### Study population and sampling

One hundred and thirty seven (137) infants from the Drakenstein community in South Africa were enrolled between May 29^th^ 2012 and May 31^st^ 2014, as part of longitudinal, prospective birth-cohort study^16^. The community is a stable, semi-urban, poor community in South Africa, receiving routine immunization with *Haemophilus influenzae* type b [Hib] and PCV13 conjugate vaccines as part of the national immunisation programme^17^. A 7-valent pneumococcal conjugate vaccine (PCV7) was introduced into the South African Expanded Program on Immunisation (EPI) in April 2009 with no catch-up immunisation but replaced by PCV13 (Prevnar^**®**^, Wyeth Pharmaceuticals Inc.) in June 2011 with provision for catch-up vaccination of unimmunised children at 18 months. PCV13 is administered in a 2+1-dosing schedule at 6 weeks, 14 weeks and 9 months of age^18^.

This study was approved by the Human Research Ethics Committee of the Faculty of Health Sciences, University of Cape Town (HREC ref: 401/2009 and 740/2013) and the Western Cape Provincial Child Health Research Committee. Written, informed consent was obtained from the participants’ parents or guardian at recruitment and annually thereafter. Enrolment of participants and all procedures were conducted in accordance with the relevant regulations and guidelines.

The details of the birth cohort study population and study design are described elsewhere^16^.

Briefly, pregnant women (>18 years), between 20 and 28 weeks’ gestation, attending antenatal care at two primary care clinics (Mbekweni and TC Newman) were enrolled and prospectively followed-up through pregnancy and childbirth (*n* = 1143 live births). NP swabs were collected from infants at birth and every alternate week during the first year of life. The subset of 137 children included in this analysis were selected consecutively from the first child completing 1 year of follow up in the birth cohort, and with adequate specimen collections (at least 23 of the 26 possible NP swabs collected). The collected NP swabs were immediately placed into 1 ml skim milk-tryptone-glucose-glycerol (STGG), transported at 4 °C to the laboratory within 2 hours of collection and frozen at −80 °C for later batch culture. Presumptive pneumococcal isolates were identified by colony morphology, α-hemolysis, optochin disk susceptibility (Oxoid, Basingstoke, UK) and confirmed using *lyt*A PCR^19^. Serotyping was performed on a single morphologically distinct pneumococcal colony per sample by sequetyping^20^ and confirmation using the Quellung method.

### Statistical analysis

Exploratory statistics were performed using STATA software (Stata Corporation, College Station, TX) and the rest of the analyses were performed using R, version 3.1.1^21^. A pneumococcal acquisition event was defined by the detection of a pneumococcal serotype for the first time in an infant, and when a pneumococcal serotype was recovered following two consecutive negative NP swab cultures for that specific serotype (Supplementary Fig. S1)^12^. A pneumococcal carriage episode, representing ongoing NP colonization, was defined as the period between acquisition and loss of the same pneumococcal serotype. Acquisition was presumed to start at the midpoint between the last of two negative NP swabs and the first positive NP swab for a specific serotype, whilst clearance was considered as the midpoint between the last positive swab and the first of two consecutive negative NP swabs for that specific serotype (Supplementary Fig. S1). Carriage duration was right censored at the last NP sampling point to account for uncertainty in how long pneumococci may have been carried after the last visit. Time to pneumococcal acquisition and carriage duration were determined by Kaplan-Meier survival estimates and recurrent colonization episodes were determined by the conditional gap-time model^22^. The model investigates time to pneumococcal acquisition on condition that the infant had previously acquired a pneumococcus. Here the survival function measures the probability of not experiencing the second event before time *t*_2_ − *t*_1_ where the individual experienced the first acquisition event at time *t*_1_, where *t*_1_ and *t*_2_ are the first and second acquisition events respectively. We report both cumulative (averaged by the number of children) and adjusted rates of acquisition and carriage duration (averaged by the number of serotypes) in order to reflect child-specific carriage dynamics and serotype-specific carriage dynamics, respectively.

### Data availability

The datasets generated during and/or analysed during the current study are available from the corresponding author on reasonable request.

## Results

One hundred and thirty-seven infants were included; including 57% (78/137) females and 55% (76/137) Black Africans, Table 1. Twenty four percent (24%, 33/137) of mothers were HIV infected, however only one child was HIV-infected due to the strong HIV prevention program. Twenty three percent (23%, 31/137) of children attended day-care. Maternal cigarette smoking was common (26%).

**Table 1:** Baseline cohort characteristics.

	Total (%) N = 137
Gender (Female)	78 (57)
Ancestry	
Black African	76 (55)
South African Colo*u*red^+^	61 (45)
Preterm delivery (<37 weeks)	20 (15)
Mode of delivery	
Normal vaginal	108 (79)
Vacuum	1 (1)
Elective caesarean	10 (7)
Emergency caesarean	18 (13)
HIV exposed*	33 (24)
Feeding practices	
Exclusively breastfed for 6 months**	29 (21)
Mixed fed at 6 months	72 (53)
Never breastfed	43 (31)
Low birth weight (<2500 g)	18 (13)
Day-care^**#**^	31 (23)
Maternal antepartum cigarette smoking	36 (26)
Household size	
Less than three people	72 (53)
Four or five people	38 (28)
More than five	10 (7)
Missing data	17 (12)

^+^Culturally and politically self-identified community comprised of Khoisan, Bantu-speaking Africans, European, and a smaller Asian genetic heritage. *Only one child born to an HIV mother was HIV positive. ^#^No information on day-care attendance was available for one child. **Data for participants with completed 6-month feeding questionnaires.

A total of 3331 NP swabs were collected (median number of swabs per infant, 25 [IQR, 23–26]). Pneumococci were isolated from 54% (1809/3331) of samples. A pneumococcal serotype was successfully assigned to 91% (1637/1809) of isolates; 9% (172/1809) were non-typeable. Immunization coverage was 100% at the scheduled 6, 14 and 40 weeks visits respectively. However, vaccination was delayed by more than 2 weeks in 6% (8/137) and 18% (24/137) of children at 6 and 40 weeks respectively.

### Acquisition and prevalence of pneumococcal carriage

**Figure 1:**
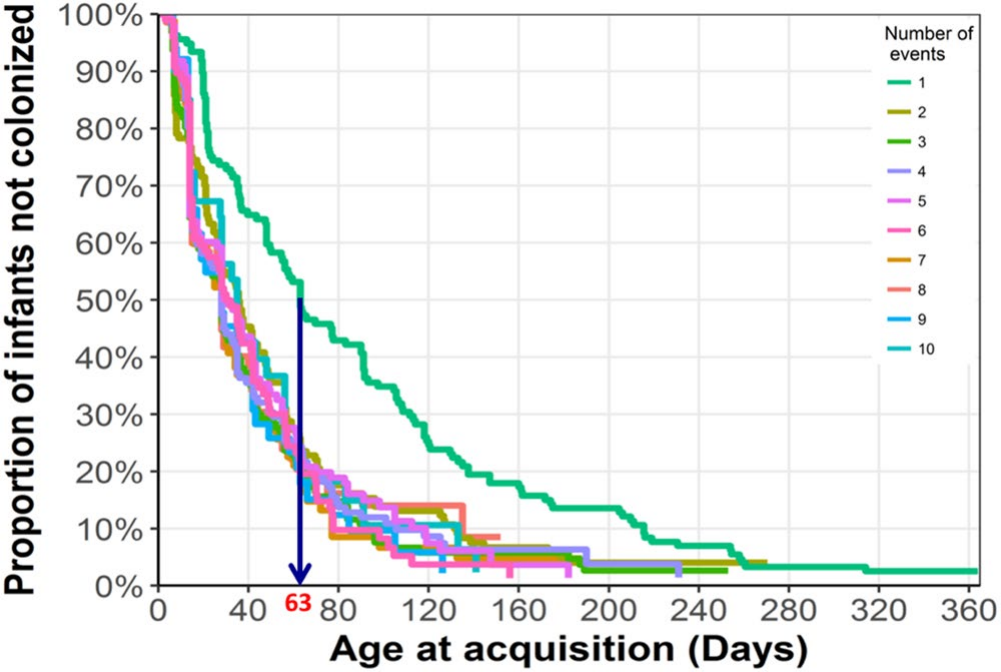
Conditional Gap model for recurrent pneumococcal acquisition events during the first year of life, n = 137 children.

Figure 1 shows the conditional-gap time model for the time-to-acquisition of first and recurrent pneumococcal carriage. All but seven (5%, 7/137) children were colonized at least once by 260 days of life. Pneumococci were not detected in three (2%, 3/137) children at any time point during the first year of life. Time to first colonization (median age, 63 days [IQR 55–90 days]) was longer than the time between first and 2^nd^ acquisition (36 days [IQR 28–47 days]) (Fig. 1 and Supplementary Table S1). Thereafter, subsequent acquisition events occurred at similar intervals. There was no difference between PCV13 and non-PCV13 serotypes in time to first pneumococcal acquisition (*p* = 0.69).

**Figure 2:**
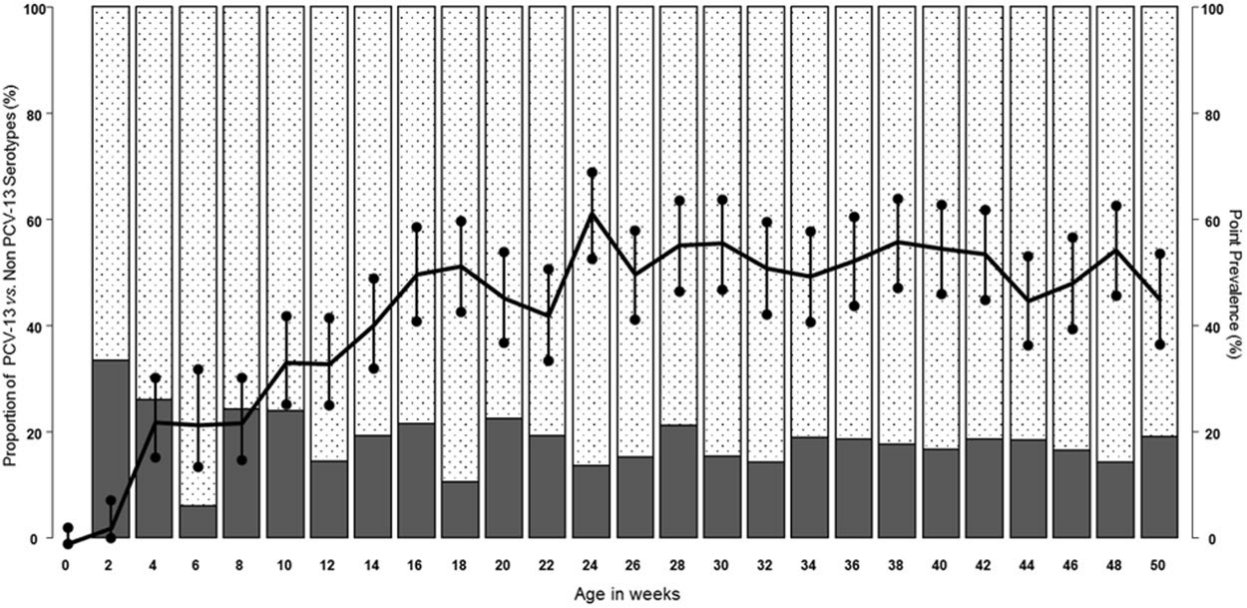
Pneumococcal carriage prevalence (right axis, black line with 95% CI at each time-point) and proportion of PCV13 or non-PCV13 pneumococcal serotypes detected at each sampling time point (left axis, solid black bars = PCV13 serotype, white checkered bars = Non-PCV13 serotypes), n = 137 children.

None of the children enrolled were colonized at birth, Fig. 2. The pneumococcal point prevalence at 2 weeks of age was 3% (95% CI 1–8%), reaching a maximum prevalence of 64% (95% CI 56–69%) at 24 weeks, then plateauing for the remainder of the first year of life (*X*^2^ test for trend *p* = 0.0001). The proportion of PCV13 serotypes remained relatively constant (~20%) over the year (Fig. 2), with no decline following each successive dose of PCV13.

### Serotype-specific acquisition rates and carriage duration

**Figure 3:**
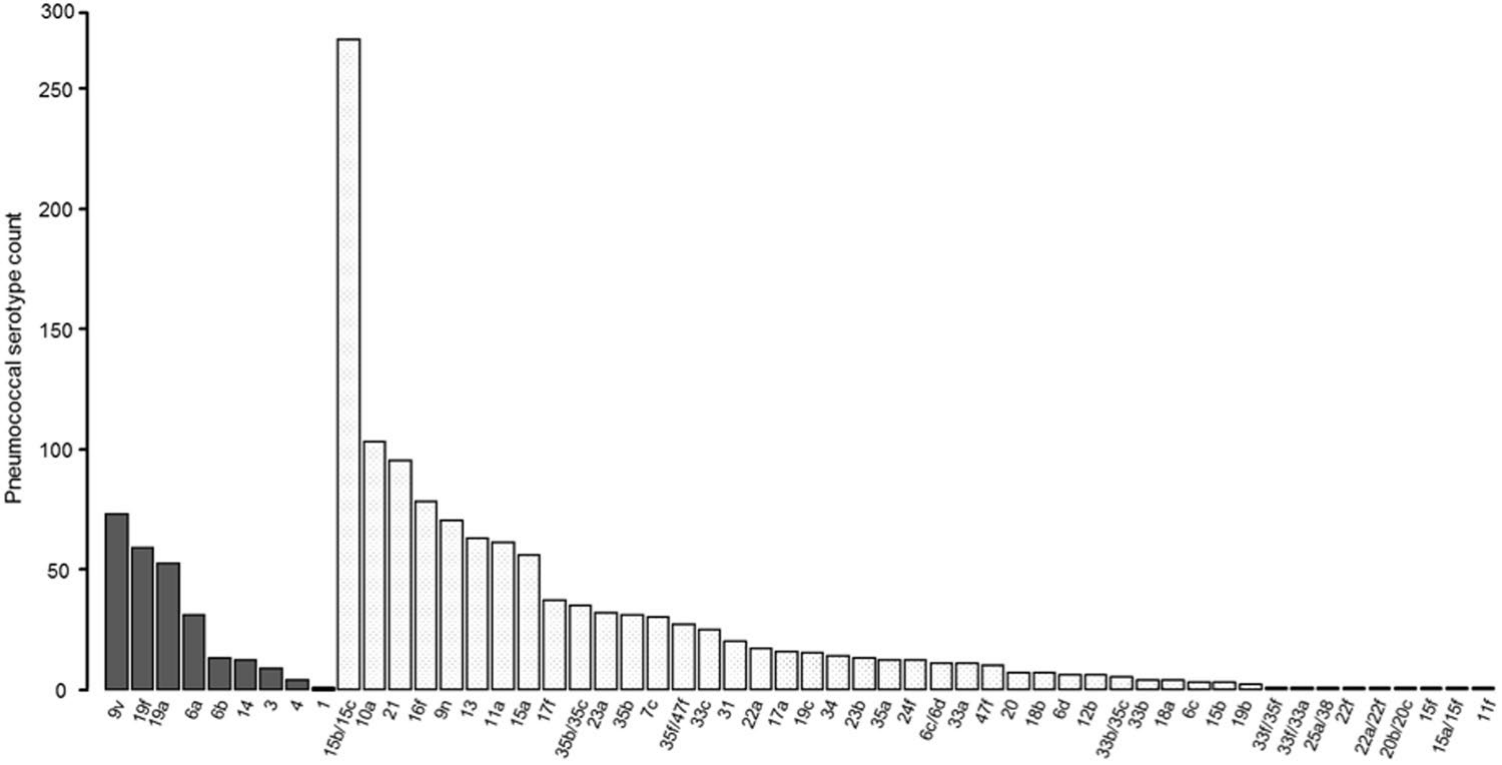
Pneumococcal serotype distribution. Black bars = PCV13 serotype, white checkered bars = non-PCV13 serotypes, n = 137 children.

Of the 48 different pneumococcal serotypes detected in this cohort, the most frequently encountered PCV13 serotypes included 9V, 19F, 19A and 6A, while those of non-PCV13 serotypes included 15B/15C, 10A, 21, 16F, 35B, 9N and 15A, Fig. 3. In total, 78% (1268/1637) of isolates were serotypes not included in PCV13.

**Figure 4:**
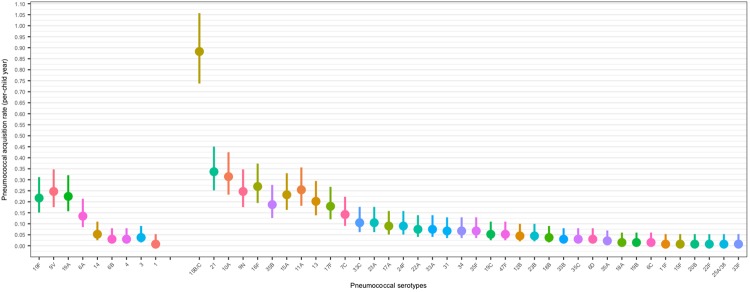
Pneumococcal acquisition rates (episodes per child year) among infants, by serotype, separated in to PCV13 and non PCV13 serotypes and then in decreasing order of acquisition rate, n = 137 children.

Serotype-specific acquisition rates ranged from 0.01 to 0.88-events/child year, with acquisition strikingly higher for serotypes 15B/15C (0.88 events/child year [95% CI 0.74–1.06]) than for any other serotype (Fig. 4 and supplementary Table S2). The acquisition rates for PCV13, non-PCV13 and non-typeable pneumococci were 0.98 events/child year (95% CI 0.82–1.16), 4.74 events/child year (95% CI 4.39–5.12) and 1.47 events/child year (95% CI 1.28–1.68) respectively. This suggests that each child experienced one new acquisition of PCV13, non-PCV13 and non-typeable pneumococci on average every 372.7 days, 77.06 days and 248.47 days respectively. When adjusted for the number of serotypes within each group, there was no difference in the average acquisition rates per serotype for PCV13 and non-PCV13 serotypes, 0.11 events/child year (95% CI 0.07–0.18) *vs*. 0.11 events/child year (95% CI 0.06–0.18), *p* = 0.95. The 9 most commonly acquired non-PCV13 serotypes were however more commonly acquired than the 9 PCV13 serotypes detected (acquisition rate 2.91 events per child year [95% CI 2.64–3.22] vs. 0.98 events per child year [95% CI 0.82–1.16]).

**Figure 5:**
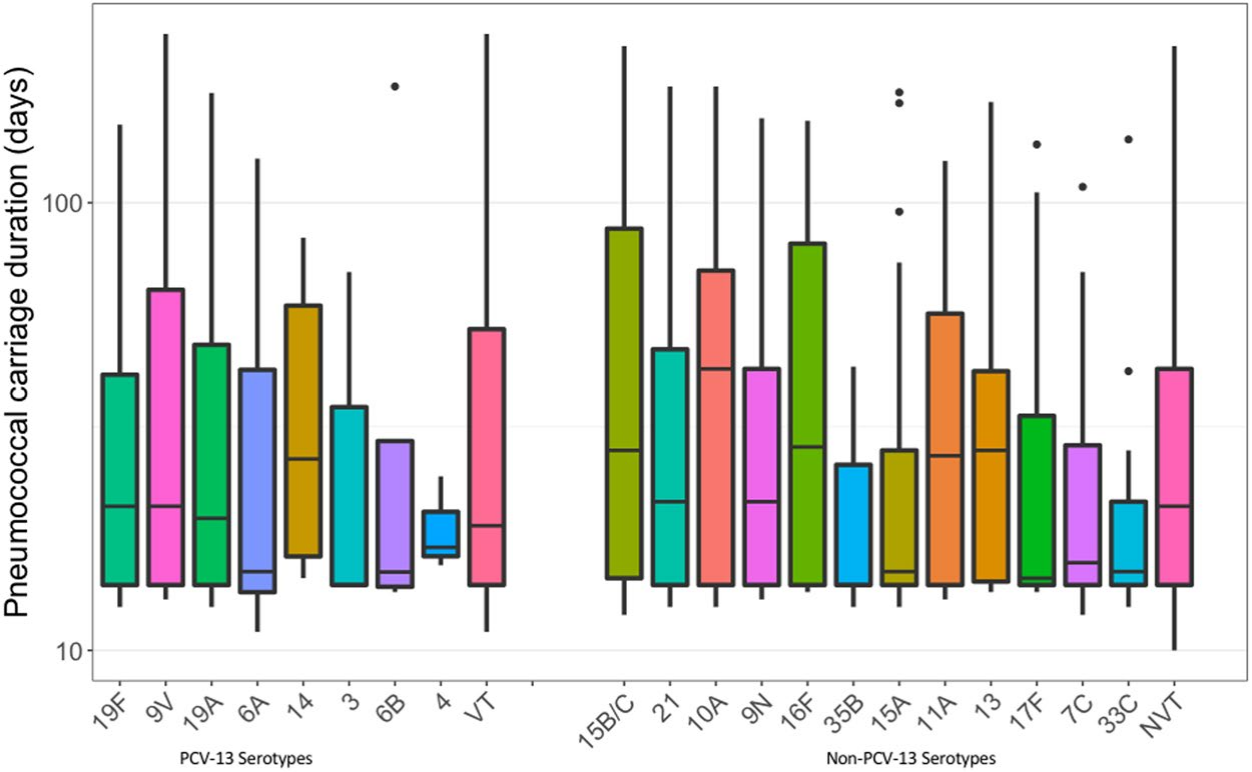
Serotype-specific duration of pneumococcal carriage (days) among infants. The “VT” and “NVT” box plots represent the average carriage duration for PCV13 and non-PCV13 serotypes respectively.

The average duration of pneumococcal carriage with a specific serotype (calculated by adding the duration of all carriage episodes for that serotype) over the first year of life was 30.28 days (95% CI 26.04–34.52). The average carriage duration varied between 13 and 65 days for the 48 different serotypes detected (Fig. 5 and Supplementary Table S2). When considering cumulative carriage duration with different serotypes, each child carried PCV13, non-PCV13 or non-typeable pneumococcal serotypes for 30.66 days (95% CI 22.3–39.01), 141.22 days (95% CI 126.61–155.84) and 30.87 days (95% CI 26.59–35.14) respectively (Supplementary Table S2). When adjusted for the number of serotypes within each group, there was no difference in the average carriage duration between PCV13 and non-PCV13 serotypes, 40.64 days (95% CI 31.92–49.36) vs. 38.58 days (95% CI 35.09–42.07), *p* = 0.67. The average carriage duration for non-typeable pneumococci was 27.39 days (95% CI 23.29–31.49).

## Discussion

This study describes longitudinal patterns of NP pneumococcal colonization over the first year of life in an intensively sampled, PCV13 vaccinated South African birth cohort. Despite the high immunization coverage, pneumococcal carriage prevalence and duration of carriage (on average one third of the first year of life) were high. Children were less likely to be colonized by PCV13 serotypes compared to non-PCV13 serotypes at all time points. The pneumococcal carriage point prevalence increased with age, reaching a maximum at 64% after 24 weeks. We showed no differences in the overall serotype-specific acquisition rates or duration of carriage between PCV13 and non-PCV13 serotypes.

The median age at first pneumococcal acquisition (63 days) is similar to that observed in Bangladeshi^14^ and Australian Aboriginal^23^ birth cohorts (50% by at 56 days and 39% by 60 days respectively), but higher than those reported for infants in other African countries, including The Gambia (24 days)^15^ and Kenya (39 days)^24^. However, both African studies were conducted prior to the introduction of PCV-7 into national immunisation schedules. These differences in age to first acquisition may reflect the impact of herd protection from PCV vaccination on force of exposure, differences in living conditions, or host susceptibility. NP sampling frequency may also affect measures of carriage; Vives *et al*. showed that pneumococci were isolated at least once from 36% *vs*. 79% of Costa Rican children who were sampled quarterly *vs*. weekly^25^. In our cohort, after the first acquisition of NP carriage, time to second acquisition was much reduced, perhaps reflecting that children are repeatedly exposed to pneumococci after reaching a certain age^26^.

Although the cumulative acquisition rates showed that children were 4 times more likely to acquire non-PCV13 serotypes than PCV13 serotypes, on average, individual PCV13 and non-PCV13 serotypes were acquired at an equivalent rate. Pre-PCV data from Kilifi, Kenya, showed higher acquisition rates for some PCV serotypes compared to our data (e.g., serotype 19F, 0.80 episodes/child-year vs. 0.22 episodes/child-year; serotype 6A, 0.66 episodes/child-year *vs*. 0.13 episodes/child-year). The converse was true for non-PCV serotypes (e.g., serotype 15B, 0.40 episodes/child-year vs. 0.88 episodes/child-year; serotype 21, 0.07 episodes/child-year *vs*. 0.33 episodes/child-year)^26^. Overall, in our PCV13 vaccinated study population, the risk of acquiring individual PCV serotypes was similar to that of acquiring non-PCV serotypes, although non-PCV13 serotypes were acquired more frequently than PCV13 serotypes.

Our current understanding of the serotype-specific immunity afforded by PCV is incomplete. In our study, ‘residual’ carriage of PCV13 pneumococci as children aged did not appear to be affected by sequential doses of PCV13. As reported elsewhere, 9V, 19F, 19A and 6A are the most predominant PCV13 serotypes in many settings with high PCV coverage^27^. Data from native American populations has shown that although PCV13 induces higher immunoglobulin G (IgG) concentrations and functional activity against 19F, the vaccine had no additional impact on 19F carriage compared to PCV7^28^. Residual 19F carriage and disease has similarly been documented in other populations that have introduced a 19F-containing vaccine^29–31^. It has been suggested that the more prevalent colonisers such as serogroups 6, 18, 19, and 23 incur lower metabolic costs associated with capsule expression and may have greater ability to form biofilms thereby resisting elimination by host mediated immune responses^32^. In addition, the continued circulation of PCV13 serotypes might be attributed to incompletely immunised older siblings or adults with waning immunity (particularly HIV-infected adults) who serve as reservoirs for transmission. Our finding that very few children were colonized by PCV13 serotypes 1 and 3, serotypes particularly associated with invasive disease, is consistent with data from elsewhere showing they are rarely detected among carriage isolates^33^.

Serotypes 15B/15C, 10A, 21, 16F, 9N, 13, 11A, 15A, 17F, 31 and 22A are currently among the most prevalent replacement, non-PCV13, serotypes globally^15,34–36^. Serotype 15B/15C has emerged as one of the predominant serotypes recovered after PCV roll out in both low and high-income countries^37–39^. Much of this increase has been linked to the clonal expansion of dominant strains^37,39,40^. Data from serially sampled communities in the United Kingdom and the United States of America have shown that serotype 15B/15C became more dominant post PCV13 implementation where initially PCV13 serotype 19A was circulating at equal proportions at baseline^37,40^. Animal models suggest that 15B/15C is equally capable of causing middle ear infections as 19A^41^. An increase in 15B/15C IPD cases have been noted from the UK and USA^42,43^. A higher valency PCV vaccine, PCV15, is currently under development, and includes all PCV13 serotypes plus 22F and 33F^44^. In our cohort, serotypes 22F and 33F were uncommon, detected in two and three children respectively. However, these two serotypes are more commonly associated with invasive infection rather than colonization.

Children are often colonized by several different pneumococcal serotypes over the first years of life, and the less immunogenic serotypes (e.g., 6, 14, 19, and 23) tend to be carried within the nasopharynx for prolonged periods of time compared to the more immunogenic strains (e.g., 3, 12, and 33)^45^. The longer duration of carriage observed in our settings for PCV13 serotypes 9V, 19F, 19A, 6A, 6B and 14 are consistent with these findings (carriage durations ranging from 30 to 56 days). Serotypes 1 and 4 were carried for shorter periods of times (carriage duration 14 days each). Non-PCV13 serotypes were generally carried for similar periods of time as PCV13 serotypes with exception of serotype 35A which was carried for 65 days with wide confidence intervals. A recent genome-wide study from Malawi has shown that recombination rates in pneumococcal lineages increase with carriage duration and size of the capsular polysaccharide^32,46^. Continued surveillance of pneumococcal carriage and invasive disease is needed to monitor the impact of targeted vaccine strategies.

Limitations of this study include the inability to detect multiple pneumococcal carriage. Serotyping was performed on a single morphologically distinct pneumococcal colony per sample. It is hard to detect serotype mixtures in this way due to the subjectivity in picking colonies and the potentially low abundance of a second serotype. We are in the process of investigating multiple pneumococcal serotypes using multiplex PCR^47^ and whole genome shotgun sequencing of total nucleic acid directly extracted from NP swabs. The clinical and public health implication of co-colonization by multiple pneumococcal serotypes is not well described and therefore warrants further investigation. Studies have suggested that multiple carriage is key in understanding microbial interactions, transfer of genetic material, impact of selective pressure on the broader microbiome as well as improving accuracy of vaccination-induced shifts in the NP ecology^48^.

We did not perform any genomic characterisation of our isolates and assumed that if an identical serotype was isolated at consecutive time points this represented the same strain. We may therefore have underestimated acquisition rates. We are currently undertaking whole genome sequence analysis of all pneumococci in the full birth cohort in order to address this issue. The lack of pneumococcal carriage or vaccination data from mothers as well as siblings living in the same household prevents detailed analysis of pneumococcal transmission patterns. Detailed analysis of risk factors for pneumococcal carriage was beyond the scope of the present study but is addressed in detail in another manuscript under review. In addition, the relatively small sample size (albeit very intensively sampled), and the small numbers of less-frequently detected serotypes detected affected the precision of some of our estimates of carriage.

In conclusion, our data show that the rate of pneumococcal acquisition and duration of carriage is serotype-specific, with residual PCV13 pneumococci still circulating despite high immunisation coverage. We detected no overall differences in time to first acquisition, acquisition rate or duration of carriage between PCV13 and non-PCV13 serotypes, however the most prevalent non-PCV13 serotypes were acquired more commonly than PCV13 serotypes, and non-PCV13 serotypes were carried cumulatively for longer than PCV13 serotypes.

## Electronic supplementary material

Supplementary information

## References

[1] Walker CLF (2013). Global burden of childhood pneumonia and diarrhoea. Lancet.

[2] Wang, H. *et al*. Global, regional, and national levels of neonatal, infant, and under-5 mortality during 1990–2013: a systematic analysis for the Global Burden of Disease Study 2013. *The Lancet*, 10.1016/S0140-6736(14)60497-9 (2014). 10.1016/S0140-6736(14)60497-9 PMC4165626 24797572

[3] Collaboration GBD (2016). Global and national burden of diseases and injuries among children and adolescents between 1990 and 2013: findings from the Global Burden of Disease 2013 Study. JAMA pediatrics.

[4] Rudan I (2013). Epidemiology and etiology of childhood pneumonia in 2010: estimates of incidence, severe morbidity, mortality, underlying risk factors and causative pathogens for 192 countries. Journal of global health.

[5] Bogaert D, De Groot R, Hermans PWM (2004). Streptococcus pneumoniae colonisation: the key to pneumococcal disease. The Lancet infectious diseases.

[6] Kllyhty H, Auranen K, Dagan R, Goldblatt D, O’Brien KL (2013). Case for Carriage. Vaccine.

[7] Bentley SD (2006). Genetic analysis of the capsular biosynthetic locus from all 90 pneumococcal serotypes. PLoS Genetics.

[8] Scott JR (2012). Impact of more than a decade of pneumococcal conjugate vaccine use on carriage and invasive potential in Native American communities. The Journal of infectious diseases.

[9] von Mollendorf C (2017). Estimated severe pneumococcal disease cases and deaths before and after pneumococcal conjugate vaccine introduction in children younger than 5 years of age in South Africa. PLoS ONE.

[10] Nzenze Sa Fau - Madhi, S. A. *et al*. Imputing the Direct and Indirect Effectiveness of Childhood Pneumococcal Conjugate Vaccine Against Invasive Pneumococcal Disease by Surveying Temporal Changes in Nasopharyngeal Pneumococcal Colonization. 10.1093/aje/kwx048 28482004

[11] von Gottberg A (2014). Effects of Vaccination on Invasive Pneumococcal Disease in South Africa. New England Journal of Medicine.

[12] Turner P (2012). A longitudinal study of Streptococcus pneumoniae carriage in a cohort of infants and their mothers on the Thailand-Myanmar border. PloS one.

[13] Gray BM, Turner ME, Dillon HC (1982). Epidemiologic studies of Streptococcus pneumoniae in infants. The effects of season and age on pneumococcal acquisition and carriage in the first 24 months of life. American journal of epidemiology.

[14] Granat SM (2007). Longitudinal study on pneumococcal carriage during the first year of life in Bangladesh. The Pediatric infectious disease journal.

[15] Hill PC (2008). Nasopharyngeal carriage of Streptococcus pneumoniae in Gambian infants: a longitudinal study. Clinical infectious diseases: an official publication of the Infectious Diseases Society of America.

[16] Zar, H. J., Barnett, W., Myer, L., Stein, D. J. & Nicol, M. P. Investigating the early-life determinants of illness in Africa: the Drakenstein Child Health Study. *Thorax*, 10.1136/thoraxjnl-2014-206242 (2014). 10.1136/thoraxjnl-2014-206242 PMC5107608 25228292

[17] le Roux DM, Myer L, Nicol MP, Zar HJ (2015). Incidence and severity of childhood pneumonia in the first year of life in a South African birth cohort: the Drakenstein Child Health Study. The Lancet. Global health.

[18] Madhi SA, Cohen C, von Gottberg A (2012). Introduction of pneumococcal conjugate vaccine into the public immunization program in South Africa: translating research into policy. Vaccine.

[19] Dube FS (2015). Comparison of a Real-Time Multiplex PCR and Sequetyping Assay for Pneumococcal Serotyping. PloS one.

[20] Leung MH (2012). Sequetyping: serotyping Streptococcus pneumoniae by a single PCR sequencing strategy. Journal of clinical microbiology.

[21] R: A language and environment for statistical computing (Vienna, Austria, 2014).

[22] Prentice RL, Williams BJ, Peterson AV (1981). On the Regression Analysis of Multivariate Failure Time Data. Biometrika.

[23] Watson K (2006). Upper respiratory tract bacterial carriage in Aboriginal and non-Aboriginal children in a semi-arid area of Western Australia. The Pediatric infectious disease journal.

[24] Tigoi CC (2012). Rates of acquisition of pneumococcal colonization and transmission probabilities, by serotype, among newborn infants in Kilifi District, Kenya. Clinical infectious diseases: an official publication of the Infectious Diseases Society of America.

[25] Vives M (1997). Nasopharyngeal colonization in Costa Rican children during the first year of life. The Pediatric infectious disease journal.

[26] Abdullahi O (2012). Rates of acquisition and clearance of pneumococcal serotypes in the nasopharynges of children in Kilifi District, Kenya. The Journal of infectious diseases.

[27] Grant LR (2013). Comparative immunogenicity of 7 and 13-valent pneumococcal conjugate vaccines and the development of functional antibodies to cross-reactive serotypes. PLoS One.

[28] Grant LR (2016). Impact of the 13-Valent Pneumococcal Conjugate Vaccine on Pneumococcal Carriage Among American Indians. Pediatr Infect Dis J.

[29] Isaacman DJ, McIntosh ED, Reinert RR (2010). Burden of invasive pneumococcal disease and serotype distribution among Streptococcus pneumoniae isolates in young children in Europe: impact of the 7-valent pneumococcal conjugate vaccine and considerations for future conjugate vaccines. Int J Infect Dis.

[30] Ladhani SN (2013). Invasive pneumococcal disease after routine pneumococcal conjugate vaccination in children, England and Wales. Emerg Infect Dis.

[31] Snape MD (2010). Immunogenicity and Reactogenicity of a 13-Valent-pneumococcal Conjugate Vaccine Administered at 2, 4, and 12 Months of Age. The Pediatric Infectious Disease Journal.

[32] Weinberger DM (2009). Pneumococcal capsular polysaccharide structure predicts serotype prevalence. PLoS pathogens.

[33] Brueggemann AB (2003). Clonal relationships between invasive and carriage Streptococcus pneumoniae and serotype- and clone-specific differences in invasive disease potential. J Infect Dis.

[34] Nzenze SA (2013). Temporal changes in pneumococcal colonization in a rural African community with high HIV prevalence following routine infant pneumococcal immunization. The Pediatric infectious disease journal.

[35] Richter SS (2014). Changes in pneumococcal serotypes and antimicrobial resistance after introduction of the 13-valent conjugate vaccine in the United States. Antimicrobial agents and chemotherapy.

[36] Steens, A., Caugant, D. A., Aaberge, I. S. & Vestrheim, D. F. Decreased Carriage and Genetic Shifts in the Streptococcus pneumoniae Population After Changing the 7-Valent to the 13-Valent Pneumococcal Vaccine in Norway. *The Pediatric infectious disease journal*, 10.1097/INF.0000000000000751 (2015). 10.1097/INF.0000000000000751 26020410

[37] Croucher NJ (2013). Population genomics of post-vaccine changes in pneumococcal epidemiology. Nat Genet.

[38] Gladstone RA (2015). Five winters of pneumococcal serotype replacement in UK carriage following PCV introduction. Vaccine.

[39] Ho PL (2015). Increase in the nasopharyngeal carriage of non-vaccine serogroup 15 Streptococcus pneumoniae after introduction of children pneumococcal conjugate vaccination in Hong Kong. Diagn Microbiol Infect Dis.

[40] Gladstone RA (2017). Pre-vaccine serotype composition within a lineage signposts its serotype replacement - a carriage study over 7 years following pneumococcal conjugate vaccine use in the UK. Microb Genom.

[41] Laufer AS (2010). Capacity of serotype 19A and 15B/C Streptococcus pneumoniae isolates for experimental otitis media: Implications for the conjugate vaccine. Vaccine.

[42] Makarewicz O (2017). Whole Genome Sequencing of 39 Invasive Streptococcus pneumoniae Sequence Type 199 Isolates Revealed Switches from Serotype 19A to 15B. PLoS One.

[43] Waight PA (2015). Effect of the 13-valent pneumococcal conjugate vaccine on invasive pneumococcal disease in England and Wales 4 years after its introduction: an observational cohort study. The Lancet Infectious Diseases.

[44] Sobanjo-ter Meulen A (2015). Safety, tolerability and immunogenicity of 15-valent pneumococcal conjugate vaccine in toddlers previously vaccinated with 7-valent pneumococcal conjugate vaccine. The Pediatric infectious disease journal.

[45] Smith T (1993). Acquisition and invasiveness of different serotypes of Streptococcus pneumoniae in young children. Epidemiology and infection.

[46] Chaguza, C. *et al*. Recombination in Streptococcus pneumoniae Lineages Increase with Carriage Duration and Size of the Polysaccharide Capsule. *MBio* **7**, 10.1128/mBio.01053-16 (2016). 10.1128/mBio.01053-16 PMC5040112 27677790

[47] Pholwat, S., Sakai, F., Turner, P., Vidal, J. E. & Houpt, E. Development of a TaqMan array card for pneumococcal serotyping on isolates and nasopharyngeal samples. *Journal of clinical microbiology*, 10.1128/JCM.00613-16 (2016). 10.1128/JCM.00613-16 PMC4922116 27170020

[48] Kwambana-Adams B (2017). Rapid replacement by non-vaccine pneumococcal serotypes may mitigate the impact of the pneumococcal conjugate vaccine on nasopharyngeal bacterial ecology. Scientific Reports.

