# Supplementary material for: Longitudinal characterization of nasopharyngeal colonization with Streptococcus pneumoniae in a South African birth cohort post 13-valent pneumococcal conjugate vaccine implementation
Source: Sci Rep. 2018 Aug 21;8:12497. doi: 10.1038/s41598-018-30345-5 (PMC6104038; doi:10.1038/s41598-018-30345-5)
Supplement: Supplementary file 1 — Supplementary information [file 41598_2018_30345_MOESM1_ESM.pdf]

Longitudinal characterization of nasopharyngeal colonization with *Streptococcus pneumoniae* in a South African birth cohort post 13-valent pneumococcal conjugate vaccine implementation

Author list:

Felix S. Dube[1,8,10*], Jordache Ramjith[2], Sugnet Gardner-Lubbe[3], Polite Nduru[6], F. J. Lourens Robberts[8], Nicole Wolter[4, 5], Heather J. Zar[6,7], Mark P. Nicol[8,9,10]

Affiliations:

[1]Department of Molecular and Cell Biology, Faculty of Science, University of Cape Town, Cape Town, South Africa,

[2]Division of Epidemiology & Biostatistics, School of Public Health & Family Medicine, University of Cape Town, South Africa,

[3]Department of Statistical Sciences, Faculty of Science, University of Cape Town, Cape Town, South Africa,

[4]Centre for Respiratory Diseases and Meningitis (CRDM), National Institute for Communicable Diseases of the National Health Laboratory Service, Johannesburg, South Africa,

[5]School of Pathology, Faculty of Health Sciences, University of the Witwatersrand, Johannesburg, South Africa,

[6]Department of Paediatrics and Child Health, Red Cross War Memorial Children's Hospital, University of Cape Town, South Africa,

[7]SAMRC Unit on Child and Adolescent Health, University of Cape Town, Cape Town, South Africa,

[8]Division of Medical Microbiology, Department of Pathology, Faculty of Health Sciences, University of Cape Town, Cape Town, South Africa,

[9]National Health Laboratory Service, Groote Schuur Hospital, Cape Town, South Africa, [10]Institute for Infectious Diseases and Molecular Medicine, Faculty of Health Sciences, University of Cape Town, Cape Town, South Africa

***Corresponding author:** Felix S. Dube, Department of Molecular and Cell Biology Building, Room 227, University of Cape Town, Rondebosch, 7701, Cape Town, South Africa.

E-mail: felix.dube@uct.ac.za

Supplemental Figure S1

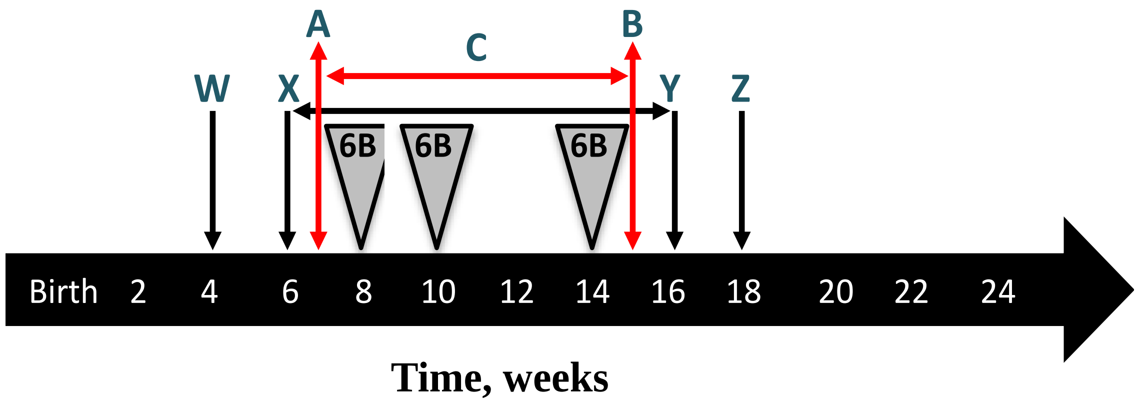

Supplementary Figure S1: Pneumococcal carriage definition. Black arrows labelled W, . Acquisition of serotype 6B pneumococcus, **A**, is defined as the midpoint between the last of two negative (W and X) swabs, in this case, **X** (at week 6) and the first positive NP swab (at week 8). Clearance of carriage, **B**, is defined as the mid-point between the last positive swab for 6B (at week 14) and the first (in this case Y, at week 16) of two consecutive negative swabs (Y and Z). Carriage duration, C, is the difference between the clearance date and date of acquisition (that is B – A), modified from Turner *et al* [13]. X, Y and Z (including week 12) represent sampling points with **negative** NP swabs

Supplementary Table S1: Conditional Gap model for median time to recurrent pneumococcal acquisition episodes.

Recurrent Acquisitions*	Number of children	Number of episodes	Median(days)	95% CI
1st	137	134	63	55 - 90
2nd	134	128	36	28 - 47
3rd	127	122	28	21 - 35
4th	122	113	28	19 - 35
5th	111	102	31	28 - 42
6th	98	89	32	22 - 42
7th	84	73	28	21.5 - 35
8th	68	56	28	15 - 42
9th	51	42	28	17 - 42
10th	40	34	35	28 - 56
11th	30	19	28	21 - NA
12th	15	11	49	27 - NA
13th	7	5	14	8 - NA
14th	5	4	15	14 - NA
15th	2	1	NA	27 - NA
16th	1	1	28	28 - NA
17th	1	0	NA	NA - NA

*1st recurrent acquisition implies 2nd carriage acquisition episode.

Supplementary Table S2: Rate of pneumococcal acquisition and duration of carriage among infants, by serotype, in decreasing order of number of acquisitions.

Serotype	Number of new Acquisitions	Rate of acquisition[b]	95% CI	Average Carriage Duration[a]	95% CI
All PCV-13	131	0.98	0.82 _ 1.16	30.66	22.3 _ 39.01
9V*	33	0.25	0.18 _ 0.35	35.69	24.95 _ 46.43
19F*	29	0.22	0.15 _ 0.31	33.29	25.01 _ 41.56
19A*	30	0.22	0.16 _ 0.32	27.65	20.17 _ 35.12
6A*	18	0.13	0.08 _ 0.21	29.5	22.19 _ 36.8
14*	7	0.05	0.03 _ 0.11	34.07	26.68 _ 41.46
6B*	4	0.03	0.01 _ 0.08	56.37	41.02 _ 71.73
3*	5	0.04	0.02 _ 0.09	29.4	23.31 _ 35.48
4*	4	0.03	0.01 _ 0.08	14.25	11.45 _ 17.04
1*	1	0.01	0 _ 0.05	14	N/A
All Non – PCV-13	634	4.74	4.39 _ 5.12	141.22	126.61 _ 155.84
15B/15C	118	0.88	0.74 _ 1.06	37.44	28.58 _ 46.29
21	44	0.33	0.25 _ 0.44	30.61	22.74 _ 38.48
10A	42	0.31	0.23 _ 0.43	39.48	29.97 _ 48.99
16F	36	0.27	0.19 _ 0.37	34.8	26.08 _ 43.53
11A	34	0.25	0.18 _ 0.36	27.2	20.53 _ 33.87
9N	33	0.25	0.18 _ 0.35	33.56	25.37 _ 41.74
15A	31	0.23	0.16 _ 0.33	28.87	20.47 _ 37.27
13	27	0.2	0.14 _ 0.29	38.07	28.85 _ 47.29
35B/35C	25	0.19	0.13 _ 0.28	21.22	16.95 _ 25.48
35B	25	0.19	0.13 _ 0.28	19.72	16.41 _ 23.02
17F	24	0.18	0.12 _ 0.27	25.79	18.9 _ 32.67
7C	19	0.14	0.09 _ 0.22	24.05	18.37 _ 29.73
23A	14	0.1	0.06 _ 0.18	34.28	26.31 _ 42.25
33C	14	0.1	0.06 _ 0.18	26.03	19.18 _ 32.89
17A	12	0.09	0.05 _ 0.16	19.79	15.8 _ 23.77
24F	12	0.09	0.05 _ 0.16	16.41	13.48 _ 19.34
22A	10	0.07	0.04 _ 0.14	27.6	20.73 _ 34.46
33A	10	0.07	0.04 _ 0.14	16.55	12.7 _ 20.39
35F/47F	9	0.07	0.04 _ 0.13	46.44	35.05 _ 57.83
31	9	0.07	0.04 _ 0.13	38.33	29.59 _ 47.06
34	9	0.07	0.04 _ 0.13	22.94	18.28 _ 27.6
19C	7	0.05	0.03 _ 0.11	31.92	25.23 _ 38.62
6C/6D	7	0.05	0.03 _ 0.11	27.5	19.75 _ 35.24
47F	7	0.05	0.03 _ 0.11	21.92	17.92 _ 25.93
23B	6	0.04	0.02 _ 0.1	34.25	24.41 _ 44.08
20	6	0.04	0.02 _ 0.1	17.75	13.99 _ 21.5
12B	6	0.04	0.02 _ 0.1	13.75	11.48 _ 16.01

18B	5	0.04	0.02 _ 0.09	21.1	16.68 _ 25.51
33B/35C	4	0.03	0.01 _ 0.08	16.75	13.84 _ 19.65
6D	4	0.03	0.01 _ 0.08	16.75	13.1 _ 20.39
33B	4	0.03	0.01 _ 0.08	13.87	11.57 _ 16.17
35A	3	0.02	0.01 _ 0.07	65	49.11 _ 80.88
15B	3	0.02	0.01 _ 0.07	14.16	11.81 _ 16.52
18A	2	0.02	0 _ 0.06	27.5	22.38 _ 32.61
6C	2	0.02	0 _ 0.06	20.5	15.64 _ 25.35
19B	2	0.02	0 _ 0.06	16.5	13.71 _ 19.28
15A/15F	1	0.01	0 _ 0.05	15	N/A
11F	1	0.01	0 _ 0.05	14.5	N/A
25A/38	1	0.01	0 _ 0.05	14.5	N/A
22A/22F	1	0.01	0 _ 0.05	14	N/A
33F/33A	1	0.01	0 _ 0.05	14	N/A
33F/35F	1	0.01	0 _ 0.05	14	N/A
20B/20C	1	0.01	0 _ 0.05	13.5	N/A
22F	1	0.01	0 _ 0.05	13.5	N/A
15F	1	0.01	0 _ 0.05	13	N/A
Nontypeable	197	1.47	1.28 _ 1.69	30.87	26.59 _ 35.14

[a]Duration of carriage in days, [b]Acquisition rates given as episodes per child year.
*Serotypes included in PCV13 vaccine formulation. In instances where there was only a single occurrence of a given serotype, the 95% CI is invalid and therefore denoted as N/A
